# Mapping soil salinity using a combined spectral and topographical indices with artificial neural network

**DOI:** 10.1371/journal.pone.0228494

**Published:** 2021-05-13

**Authors:** Vahid Habibi, Hasan Ahmadi, Mohammad Jafari, Abolfazl Moeini

**Affiliations:** 1 Department of Natural Resources and Environment, Science and Research Branch, Islamic Azad University, Tehran, Iran; 2 Faculty of Natural Resource, University of Tehran, Karaj, Iran; Universidade Federal de Uberlandia, BRAZIL

## Abstract

Monitoring the status of natural and ecological resources is necessary for conservation and protection. Soil is one of the most important environmental resources in agricultural lands and natural resources. In this research study, we used Landsat 8 and Artificial Neural Network (ANN) to monitor soil salinity in Qom plain. The geographical location of 72 surface soil samples from 7 land types was determined by the Latin hypercube method, and the samples were taken to determine the electrical conductivity (EC). Thirty percent of the data was considered as a validation set and 70% as a test set. In addition to the Landsat 8 bands, we used spectral indices of salinity, vegetation, topography, and drainage (DEM, TWI, and TCI) because of their impacts on soil formation and development. We used ANN with different algorithms to model soil salinity. We found that the GFF algorithm is the best for soil salinity modeling. Also, the TWI topography index and SI5 salinity index and NDVI vegetation index had the most effect on the outputs of the selected model. It was also found that flood plains and lowlands had the highest levels of salinity accumulation.

## Introduction

Soil quality monitoring is a preventive tool for protecting natural environments and fragile ecosystems in arid lands. Land degradation involves different aspects of the degradation of natural resources. One of the manifestations of land degradation is soil salinization [1]. Today, soil salinity along with other natural disasters, such as floods and earthquakes, is becoming more visible and important to researchers. In addition to the climatic problems, Iran’s arid lands are affected by two major phenomena of desertification and soil salinization, which degrade farmlands and rangelands [2]. Due to soil salinity, soluble salts accumulate within the range of root development or soil profile and the ecological potential and biomass production is reduced, so that the soil cannot support forests, pastures, and rangelands, and eventually deserts expand [3]. In order to protect resources for future use, they must be monitored. Soil quality monitoring is a designed program, and continuous monitoring includes direct sampling, control, the study of existing changes and analysis of before and after data [4]. Soil salinity monitoring is the study of soil salinity changes at spatial and temporal resolutions [5]. The determination of saline and non-saline lands depends on the experience of the technicians their expertise and understanding of soil and environment relationships. In recent decades, the combination of information from satellite imagery and small-scale sampling has become prevalent [6]. Many researchers have used this technique to extract soil parameters and mapping [7]. Soil digital mapping involves a set of computer calculations to predict the distribution of different soil parameters in a geographical range, which is used in the form of a simpler solution for creating spatial-temporal data of physical and chemical parameters of soil with high spatial resolution capabilities [8]. Many researchers have evaluated different sensors’ capabilities and spectral indices for more precise mapping [9–11]. Artificial neural networks are one of the computational methods for machine learning, data visualization, and ultimately applying trends to estimate the outputs of complex systems in artificial intelligence systems [12, 13]. In this method, observational data is used to train the model, then the model predicts and simulates with proper accuracy [14]. According to intrinsic relationships between the variables, a linear or nonlinear model is established between the independent and dependent parameters [15–19].

Taghizadeh-Mehrjardi et al. [2] used Landsat data and topographic indices to produce a 1m depth three-dimensional salinity map. They used a regression tree to map soil salinity. They showed that with increasing depth, the prediction accuracy decreased by more than 50%. For example, the coefficient of determination, R^2^ of the model at first 15 cm of soil was 0.78, which decreased to 0.11 at a depth of 100–160 cm. Mousavi et al. [20] used salinity and vegetation spectral indices to verify the correlation of the Electrical Conductivity (EC) parameter with spectral indices. They compared artificial neural network (ANN) and multilinear regression (MLR) salinity prediction models and concluded that ANN had 46 percent higher accuracy.

Shahabi et al. [21] modeled soil salinity using ANN, Ordinary Kriging, and multiple regression methods. They used environmental parameters such as altitude, geographic aspect, slope length, TWI index, and NDVI spectral parameters. Finally, they stated that ANN had smaller MSE and higher R^2^ than two other models. The purpose of this study was to investigate the geographical differences of soil salinity with a spatial resolution of 30 m, using ANN and auxiliary data. In other words, the topography is a primary factor that controls the spatial variability of hydrological processes and spatial distribution of moisture. Therefore, in addition to spectral data, topographic indices were used to investigate the effect of topography in salinity mapping. Also, in order to study different neural network algorithms and determine the optimal algorithm to predict salinity in the study area in the present and future years, we compared different algorithms. Due to the high cost of sampling, we tried to use the hyper cubic technique to determine the sampling points to select the geographic location of a small group of soil samples with the highest accuracy.

## Materials and methods

**Fig 1:**
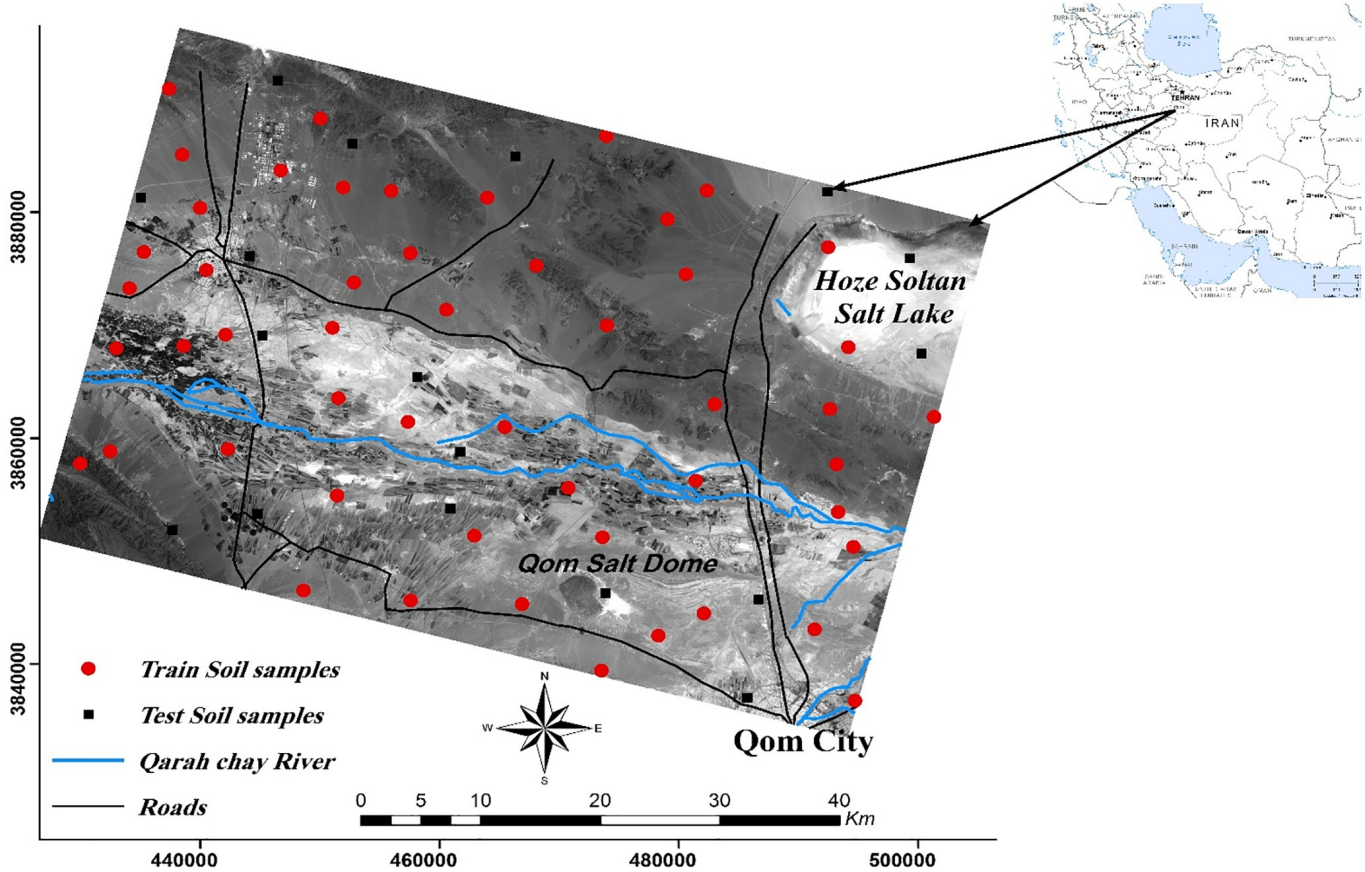
The study area bounded by the city of Qom to the southwest, by a salt dome to the south and by the Hoze-Soltan playa to the northeast.

Qom plain is located in the North and Northeast of Qom, Iran [Fig 1]. Since this area is adjacent to Ghare Chay River and due to its proximity to the capital of Iran, its crop and fruit production is very important. The plain is bounded to 34 ◦ 37’-34 ◦ 56’N and 50 ◦ 31’-51 ◦ 02 E and it covers approximately 3290 km2.

**Fig 2:**
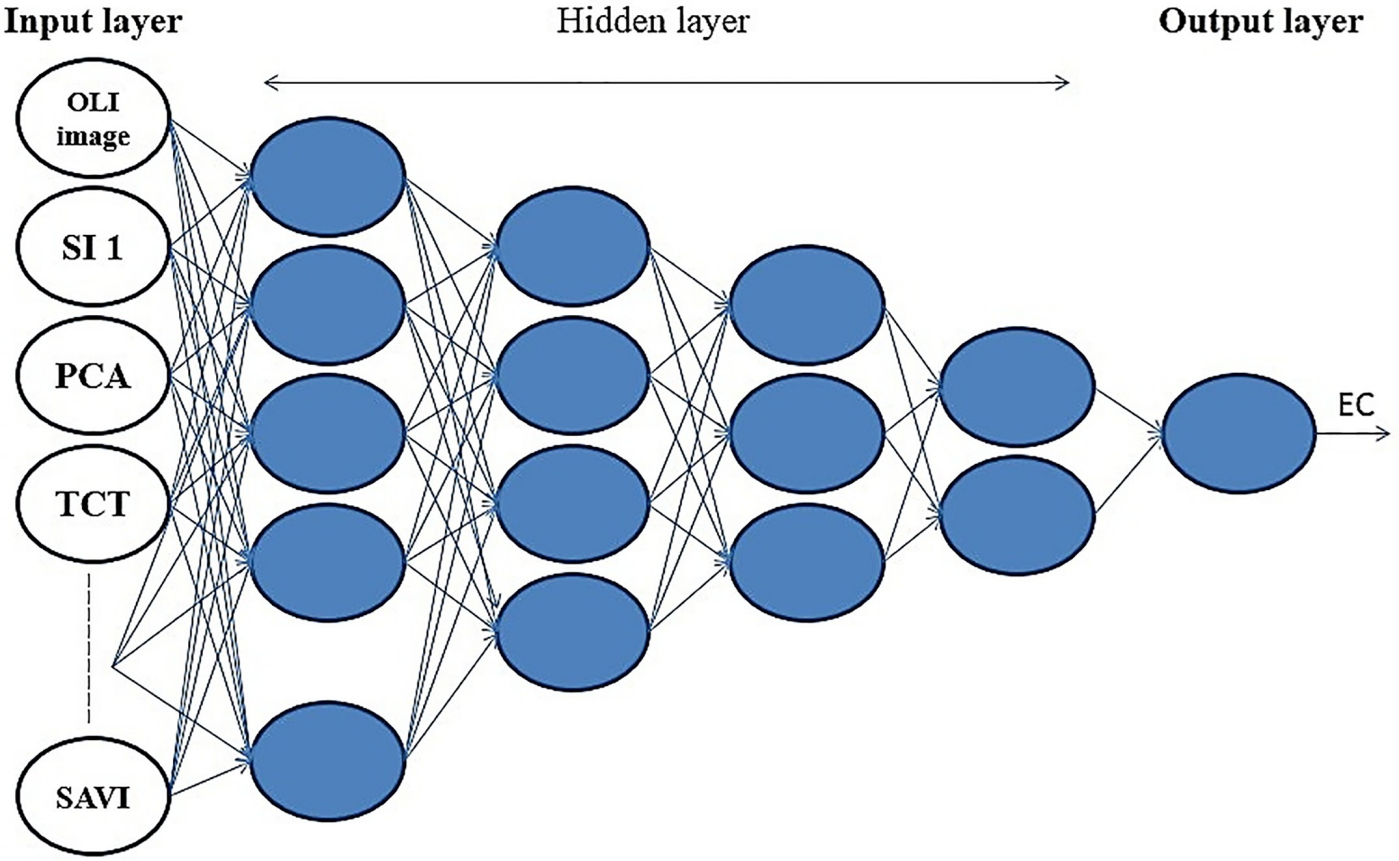
Schematic of the MLP model used in this study, including one input/target layer and four hidden layers.

In addition to the Landsat 8 data, nonlinear ANN was used for spatial modeling. The ANN is a computational approach introduced to model the neurons’ function in living tissues. McCulloch and Pitts [22] proposed a mathematical analysis for ANN from algorithms and applied mathematics called Threshold Logic to it. Choosing the right number of neurons and layers provide a better model of the ANN [23]. Network models, Multilayer Perceptron’s (MLPs), Generalized Feed Forward networks (GFF), Modular Neural Networks (MNN), Radial basis function (RBF) trained by Momentum Optimization Algorithm (MO) and Levenberg-Marquardt algorithm (LM) Learning Rule, Sigmoid Axon and Tanh Axon as transfer function were selected and compared. Five hidden layers were used to regulate the weights of neurons to achieve the desired output. According to trial and error, a replication (Epoch) of 100 was used to create the neural network. We also considered 30% of the soil EC as a validation set and the rest (70%) for a testing set. As Fig 2 shows, the MLP model has three layers; input layer, hidden layer, and output layer. Except for input nodes, each node is a neuron that uses a nonlinear activation function.

In this research, we allied the Latin hypercube approach to determine the location of samples. The Latin hypercube method is based on the stratified sampling method [24]. In this method, auxiliary variables are categorized into uniform categories. In the Latin hypercube method, the user decides to use a specific number of samples. Based on this technique, 72 samples were selected from 0 to 30 cm of the soil surface and sent to the soil laboratory for the measurement of the EC saturated extract. In the soil laboratory, the sample dried, ground and sieved with a 2-mm sieve. The electrical conductivity of saturated extract measured with EC meter and the values are corrected for 25 °C. All 72 soil samples are classified, according to Bennett and Barrett-Lennard’s (2013) method [25]. This method uses the EC level and limitation of plant growth.

Auxiliary data such as Landsat 8 satellite image (Year 2016) used and spectral/topographical indices extracted (S1 File). Digital soil mapping relies on empirical relationships between soil properties and their environmental factors such as soil, climate, organisms, topography, native materials, and their location (Tagizadeh et al. [2]). Looking at both factors, which are affecting soil salinity distribution, and the availability or feasibility of preparing such spatial environmental information, the auxiliary data are selected according to Table 1. The topography is one of the most important factors which controls the spatial pattern of soil properties and variations and hydrological processes. The TWI is a useful tool for describing the moisture conditions of an area and approximates the surface saturation and spatial distribution of soil moisture. TWI assumes that the groundwater slope equal to the slope of the ground. This index is usually used to quantify topographic control of hydrological processes. The following formula is used to calculate the index. Also Elevation parameters are influenced by morphological Facieses of the area which reflects elevation changes. Therefore, the suitable elevation units (TCI) parameter (introduced by Bohner et al.) extracted.

$$TWI=ln\left(\frac{A_{s}}{tan\beta }\right)$$ (1)

Where β is the local slope (radians) and A_s_ is the contributing area of upstream (m2).

**Table 1:** The indices used in this study.

*Index*	*Band ratio/Formula*	*References*
SI1	$\sqrt{B\times R}$	[26]
SI2	$\sqrt{G\times R}$	[26]
SI3	*B* × *R*	[27]
SI4	$\sqrt{\left(G^{2}+R^{2}+{NIR}^{2}\right)}$	[26]
SI5	$\frac{B}{R}$	[28]
SI6	$\frac{R\times NIR}{G}$	[29]
SI7	$\frac{G\times R}{B}$	[30]
SI8	$\frac{R\times B}{G}$	[28]
BI	$1+\frac{\left(R+SWIR)-(NIR+B\right)}{\left(R+SWIR)+(NIR+B\right)}$	[31]
MSAVI	$(NIR+0.5)-\frac{\sqrt{{\left(2\times NIR+1\right)}^{2}-8\times (NIR-2\times R)}}{2}$	[32]
SAVI	$\left(1+\text{L})\times (\frac{NIR-\text{R}}{NIR+\text{R}}\right)$	[33]
NDVI	$\frac{\left(NIR-R\right)}{\left(NIR+R\right)}$	[34]
NDSI	$\frac{\left(R-NIR\right)}{\left(R+NIR\right)}$	[27]
VSSI	2 × *G* − 5 × (*R* + *NIR*)	[35]
Br	b2 × 0.3029 + b3 × 0.2786 + b4 × 0.4733 + b5 × 0.5599 + b6 × 0.508 + b7 × 0.1872	[36]
Gr	b2 × −0.2941 + b3 × −0.243 + b4 × −0.5424 + b5 × 0.7276 + b6 × 0.0713 + b7 × −0.1608	[36]
We	b2 × 0.1511 + b3 × 0.1973 + b4 × 0.3283 + b5 × 0.3407 + b6 × (−0.7117) + b7 × (−0.4559)	[36]
PCA1-3	Extracted of OLI Bands	-
TWI	$\text{TWI}=\text{ln}(\frac{{\text{A}}_{\text{s}}}{\text{tan}\beta })$	[37]
DEM	Digital Elevation Model	-
TCI Low	Terrain Classification Index for Lowlands (SAGA GIS Software)	[38]

*B, G, R, NIR, SWIR, TIR, and MIR are Blue, Green, Red, Near Infra-Red, Short Wave Infra-Red, Thermal Infra-Red, and Middle Infra-Red bands, respectively. SI 1–8: Salinity Indexes, NDSI: Normalized Difference Salinity Index; and NDVI: Normalized differential vegetative index. MSAVI: Modified Soil Adjusted Vegetation Index, BI: Bare Soil Index, Br: Brightness, Gr: Greenness, We: Wetness, PCA: Principal component analysis; TWI: topographic wetness index, TCI: Terrain Classification Index for Lowlands.

The prediction performance of both sets (training and testing) is evaluated by the coefficient of determination (R^2^) and mean squared error (MSE). The calculation formula is:

$$R^{2}=\left[\frac{\sum_{i=1}^{n}(Z_{o}-\overset{}{{\overset{-}{Z}}_{p})(Z_{p}}-\overset{}{{\overset{-}{Z}}_{p})}}{\sum_{i=1}^{n}{\left(Z_{o}-{\overset{-}{Z}}_{o}\right)}^{2}(Z_{p}-\overset{}{{\overset{-}{Z}}_{p}{)}^{2}}}\right]$$ (2)

$$MSE=\frac{1}{N}\sum_{i=1}^{n}(Z_{o}-Z_{p}{)}^{2}$$ (3)

Where Z_p_ is predicted, and Z_o_ is observed, data ${\bar{Z}}_{\text{p}}$, and ${\bar{Z}}_{\text{o}}$ show the average of observed and predicted values, respectively, and N is the number of observations. MSE is the average of the squares of the difference between predicted values by the model and measured values. The coefficient of determination (R^2^) establishes a linear correlation between measured values and simulated ones by model.

## Results and discussion

**Fig 3:**
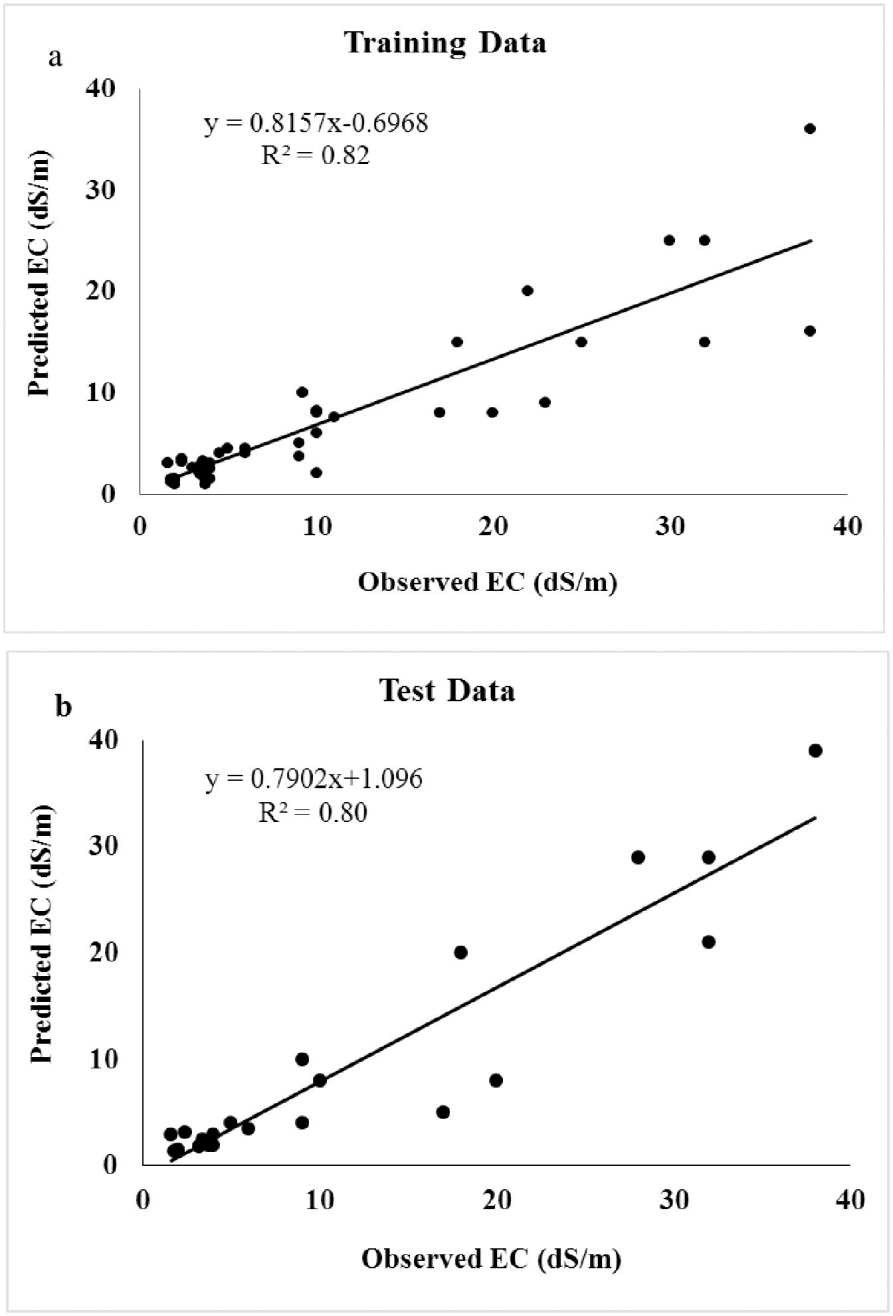
Scatter plots of the observed and estimated soil salinity with the training (a) and testing periods (b) with the selected algorithm, The R^2^ is 0.82 and 0.80 respectively.

**Fig 4:**
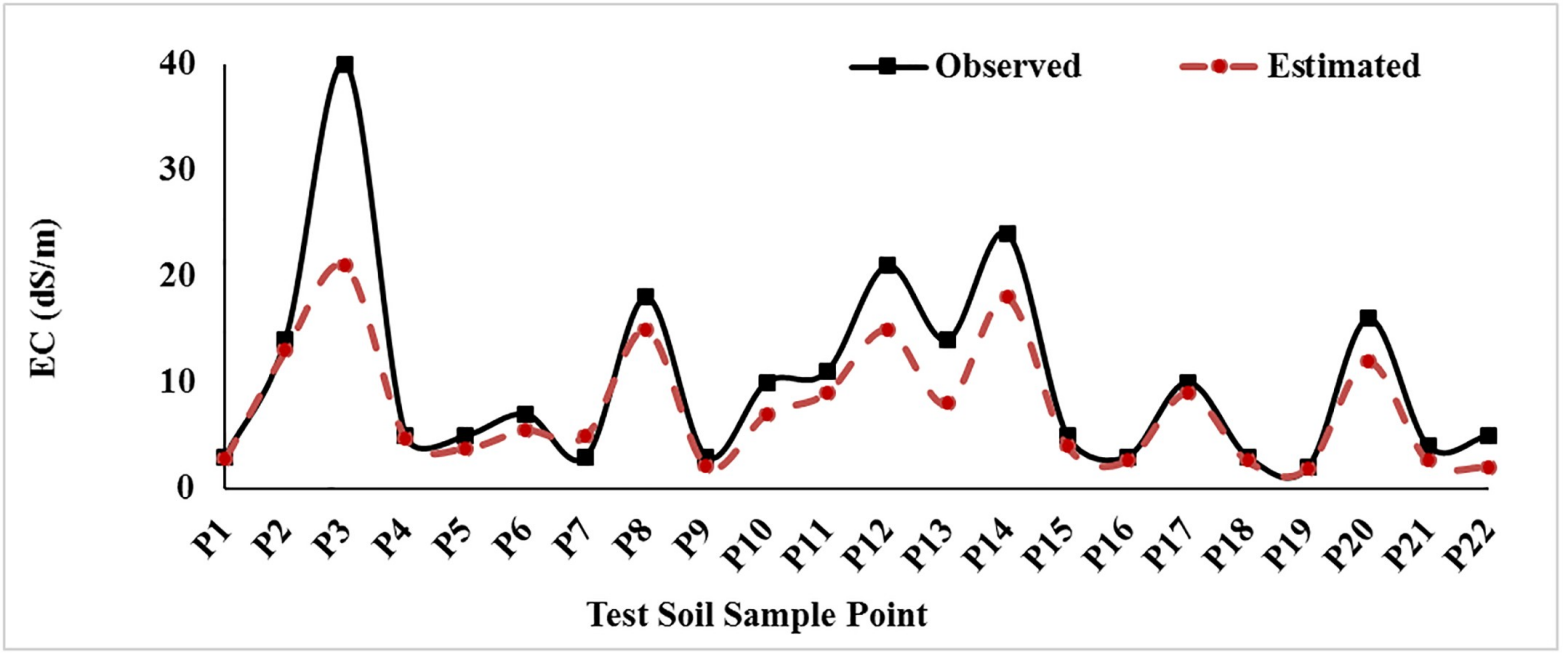
Comparison of estimated and observational EC in the selected data for testing the selected ANN model. In sample P3, the maximum difference of estimated EC and observed EC was found.

Laboratory results showed that the EC of 72 soil samples varies from 1 to 39.7 dS / m, with a coefficient of variation (CV) of 63%. CV indicates the high variability of salinity in the region. We applied ANN on 4 networks using trial and error and predetermined parameters and compared the results to find the optimum network. Table 2 shows the 4 networks of soil salinity during training and testing stages. According to the results of four ANN models (Table 2), each model was compared with two different algorithms and each algorithm with two Transfer methods. The model with the highest R^2^ value in both the Train and Test data series and also the lowest MSE value was selected as the best appropriate model. As Table 2 shows as the accuracy of the model increases, the R^2^ statistic increases, and consequently the MSE decreases. Therefore, the best model has the maximum R^2^ and the lowest MSE. The GFF algorithm, according to R^2^ and MSE statistics, the best way to prepare soil salinity map is ANN in Sharif Abad plain. Also Fig 3 shows scatter plots of the observed and estimated soil salinity at training and test period GFF algorithm. To assess the EC prediction status, we used the test dataset and mapped the observed and estimated data. As Fig 4 shows observational and estimated amounts, it was found that the ANN model satisfies in most cases the EC amount less than the real value. It is evident in the amounts of EC greater than 20 ds/m, and it is more evident in EC greater than 32 ds/m.

**Table 2:** Comparison of four models developed in training and testing stages.

Model	Algorithm	Transfer	Train		Test	
			R^2^	MSE	R^2^	MSE
**RBF**	**LM**	Tanh Axon	0.17	4.5	0.16	4.7
		Sigmoid Axon	0.16	3.1	0.15	3.3
	**MO**	Tanh Axon	0.48	2.3	0.46	2.5
		Sigmoid Axon	0.49	2.3	0.45	2.7
**MLP**	**LM**	Tanh Axon	0.48	1.5	0.45	1.7
		Sigmoid Axon	0.62	1.3	0.60	1.5
	**MO**	Tanh Axon	0.77	1	0.73	1.1
		Sigmoid Axon	0.67	1.5	0.65	1.7
**GFF**	**LM**	Tanh Axon	0.47	3.1	0.41	3.9
		Sigmoid Axon	0	6.4	0	7.1
	**MO**	Tanh Axon	0.82	1.2	0.8	1.5
		Sigmoid Axon	0.14	1.8	0.11	2.6
**MNN**	**LM**	Tanh Axon	0.44	1.8	0.36	2.6
		Sigmoid Axon	0.54	2.8	0.49	3.2
	**MO**	Tanh Axon	0.41	3.1	0.39	3.7
		Sigmoid Axon	0.65	1.8	0.61	2.3

**Fig 5:**
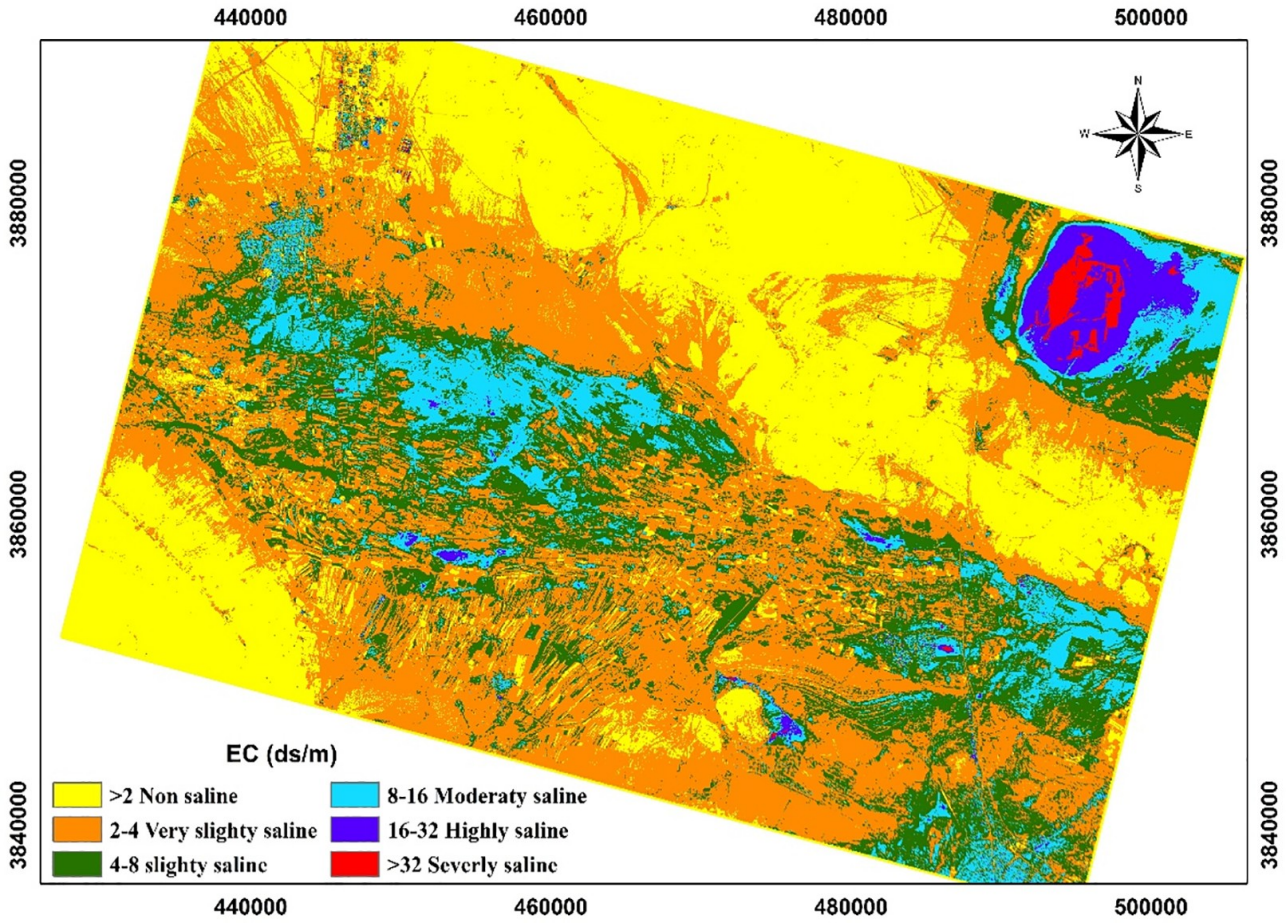
Soil salinity map of the study area using an artificial neural network, as can be seen, the highest salinity level found in the Hoze-Soltan playa in the northeast of the study area.

The map of Sharif Abad plain prepared based on the selected algorithm (Fig 5) is clearly verifiable. Around the Hoze Soltan Playa, northwest of our study area, according to the analysis of samples soil EC is more than 32 ds/m, while in the map it has a range of EC 16–32 ds / m, indicating the error of estimation by the neural network model when EC is greater than 32 ds/m. According to the map (Fig 5), as we move from the west to the east of the region and the south and north to the center and southeast of the study area, the salt accumulation in the soil becomes more evident. The highest level of integration with high salinity is in the northeast and adjacent to the Hoze Soltan Playa.

**Fig 6:**
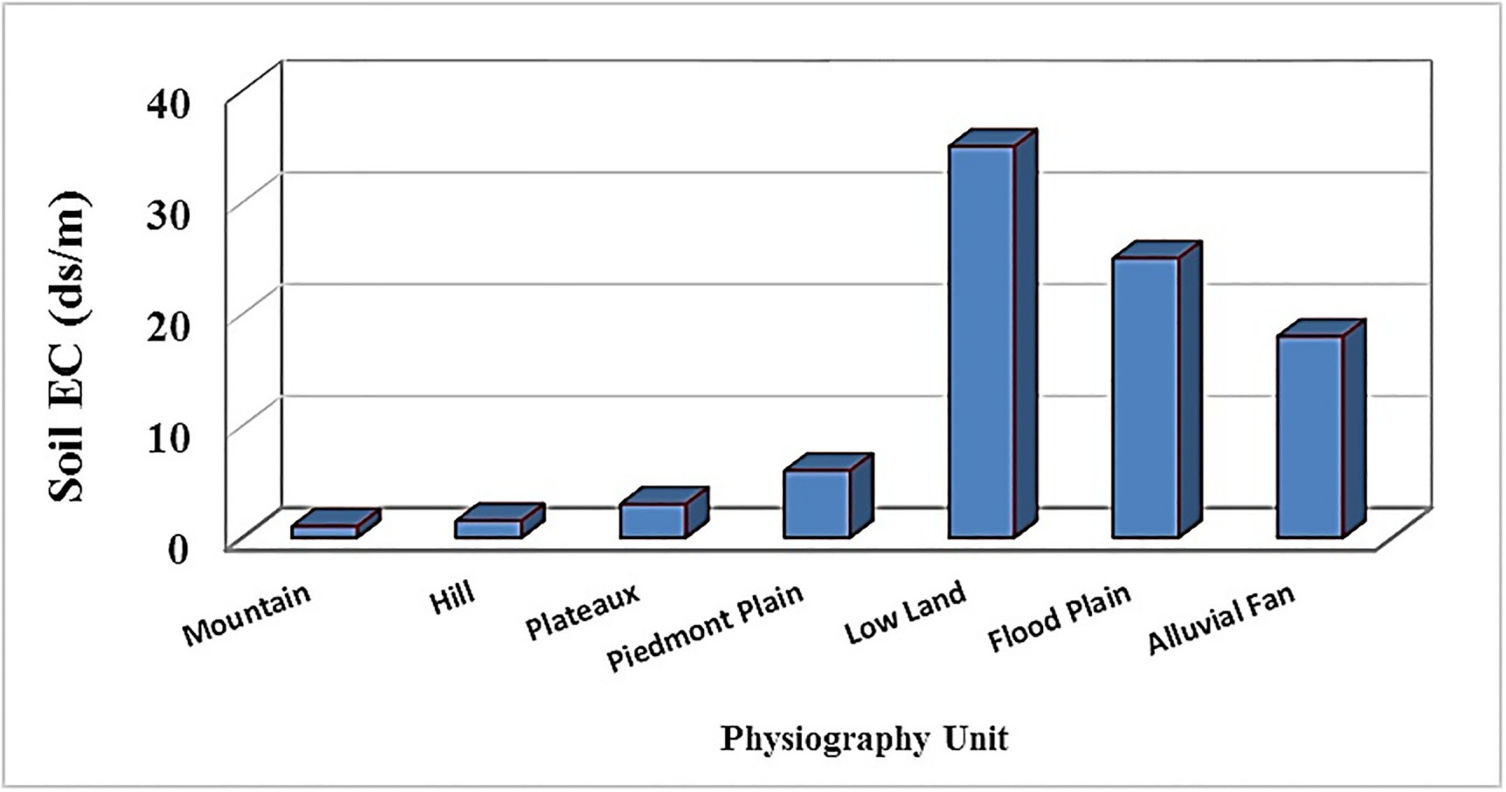
Soil salinity mean value in physiography units of the study area, Lowlands are distinguished by maximum observational salinity and mountains are minimum observational salinity.

According to Fig 5, the maximum amount of salinity is observed in the central and northeastern part of Sharif Abad plain, due to its low slope and concave topography, salt is deposited from uplands. These areas with high salinity are a type of land called plain and flood plains. The results of one-way analysis of variance (ANOVA) (Fig 6) showed significant differences among seven land types, and the results of Duncan test showed that the soil salinity level in lowlands and flood plain was more than other units. Since most of the lands in this region are mainly in the central and northeastern regions with a low gradient and concave topography, so the salts of the upper parts are washed and accumulated and caused greater salinity than other land types.

Physiographic units of the mountains, hills, plains, and plateaus are mainly located on high and steep lands, so they have less salinity. The land type of alluvial plain has less salinity than lowlands and flood plains due to a higher slope and suitable drainage network. Looking at the geology of the study area, we found that due to soluble marls and quaternary clay formation (Q_c_) in the mainstream routes of flood plains unit and transportation of salts, this unit suffers from high salinity.

**Fig 7:**
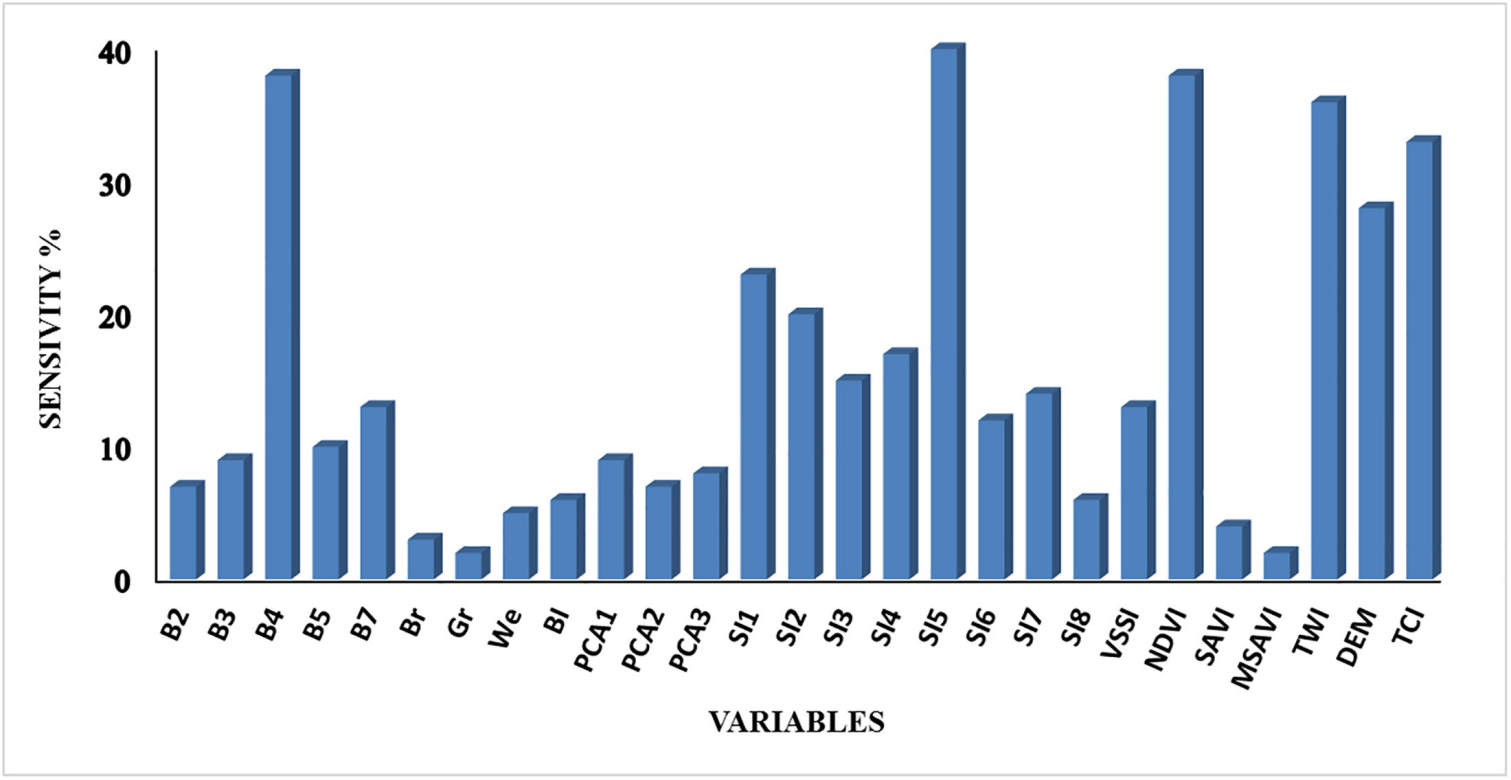
Results of variables’ importance to estimate soil salinity based on the analysis. The greenness variable has the minimum effect in the selected model and the salinity index 5 variable has the maximum effect.

The sensitivity analysis showed that the auxiliary variables of salinity index SI5, NDVI, TWI, TCI, band 4, DEM, and SI1 had the highest effect on soil salinity prediction in the study area of Sharif Abad plain (Fig 7). It indicates that in the study area, topography and vegetation cover are the greatest influential factors in soil formation and distribution because the topography and vegetation affect soil salinity. Our findings are in accordance with Bagheri et al. [13] and Shahabi et al. [21]. As Shahabi et al, selected the spectral and wetness variables and applied them as covariates in the ANN model, the accuracy of the prediction was 62%, while the selection of such variables in this study enables the mapping with an accuracy of 82%. Although Bagheri et al. used different ANN algorithms with a different number of variables. They predicted soil parameters with an accuracy of 88%. Interestingly, both studies confirm the role of DEM in increasing prediction accuracy.

The present study, like those of Ebrahimi et al., [16] Hoseini et al., [17] Roustaei et al., [18] Taghizadeh-Mehrjardi et al., [12] confirms the ability of an ANN to model the chemical and physical parameters of the soil. According to Ebrahimi et al. and Roustaei et al., studied soil physicochemical properties and showed that among ANN and MLR methods, the ANN method has an accuracy of 76% and 56% respectively. However, in the present study, the coefficient of variation of the accuracy of different algorithms is high and some algorithms showed 40% variations. According to Hoseini et al, the accuracy of the R^2^ statistic was 99% using the LM algorithm, whereas in the current study, the accuracy was 17% smaller. Also, the proposed algorithm of this study is different than Hoseini et al.

Also, Akramkhanov and Vlek [15] and Ghorbani et al. [7] emphasized that the neural network method is preferable to other nonlinear methods for mapping soil surface salinity. They proposed the use of the MLP algorithm for soil salinity modeling. Akramkhanov and Vlek reported salinity prediction with R^2^ = 0.83 and Ghorbani et al. showed an accuracy of 85%. Their studies in terms of the proposed method and the accuracy of the results are almost consistent with the present study and the only difference is the proposed algorithm.

The findings of Mohamed et al. [11] and Morgan et al. (2018) also confirmed the use of visible and infrared VIS-NIR bands along with the neural network to map soil salinity, which is in agreement with our results. Morgan et al. combined NDVI spectral indices with ANN and presented the salinity map with an accuracy of 94%.

Jin et al. [5] emphasize the use of spectral indices from satellite images to provide a salinity map as the most effective and important desertification factors. They acknowledged that the use of these indices detects soil salinity and can determine the type of salinity.

## Conclusions

The central sections of Sharif Abad plain with low elevation and vegetation cover has the highest amount of soil salinity. The best auxiliary variable for soil salinity prediction is the salinity index. The powerful correlation among soil data and auxiliary data can affect the accuracy and precision of the model. We found that the use of artificial neural network models, DEM, TCI, and TWI indices, which are affected by landform, lithology, and soil formation factors as non-explicit data can produce maps with higher precision. Also, results showed that the GFF algorithm, according to R^2^ and MSE statistics, is the best way to prepare soil salinity map ANN in Sharif Abad plain.

Among applied algorithms in four models (MNN, GFF, MLP, RBF), the MO algorithm had higher accuracy than the LM algorithm is similar transfers. According to LM algorithm characteristics, it can provide an accurate pattern with the least differences between observed and estimated values. On the contrary, increasing epochs expand the differences between the former epoch and the current epoch. The auxiliary data such as topographic indices, salinity, and land cover indirectly show the soil salinity and assist in anticipating the differences between primary and final estimation by the LM algorithm.

We found that the ANN model in most cases satisfies the EC amount less than the real value. It is evident in the amounts of EC greater than 20 ds/m, and it is more evident in EC greater than 32 ds/m. Our results showed that the salinity index could be effective in soil salinity mapping.

Since there were no qualitative/quantitative data from the study area or similar adjacent regions, we used this one set of experimental data and Landsat 8 images. To compare and understand soil salinity mapping algorithms, we recommend comparative studies using similar algorithms in various regions.

## Supporting information

S1 File The values of laboratory results of soil samples and indices extracted for the study area. B, G, R, NIR, SWIR, TIR, and MIR are Blue, Green, Red, Near Infra-Red, Short Wave Infra-Red, Thermal Infra-Red, and Middle Infra-Red bands, respectively. SI 1–8: Salinity Indexes, NDSI: Normalized Difference Salinity Index; and NDVI: Normalized differential vegetative index. MSAVI: Modified Soil Adjusted Vegetation Index, BI: Bare Soil Index, Br: Brightness, Gr: Greenness, We: Wetness, PCA: Principal component analysis; TWI: topographic wetness index, TCI: Terrain Classification Index for Lowlands.
(XLS)
